# Phase I study of aflibercept in combination with docetaxel in Japanese patients with advanced solid malignancies

**DOI:** 10.1007/s10637-022-01267-x

**Published:** 2022-06-30

**Authors:** Yu Sunakawa, Keishiro Takahashi, Osamu Kawaguchi, Nobuyuki Yamamoto

**Affiliations:** 1 Department of Clinical Oncology, St. Marianna University School of Medicine, Kawasaki, Japan; 2 Research & Development, Sanofi, Tokyo, Japan; 3 Biostatistics & Programming, Sanofi, Tokyo, Japan; 4 Third Department of Internal Medicine, Wakayama Medical University Hospital, Wakayama, Japan

**Keywords:** Aflibercept, Docetaxel, Dose escalation, Japanese, VEGF Trap

## Abstract

Angiogenesis is a hallmark of cancer development. This study sought to determine the recommended dose of aflibercept, a recombinant fusion protein targeting VEGF-A, VEGF-B and placental growth factor (PlGF), combined with docetaxel in Japanese patients with advanced solid malignancies. This phase I study was planned to include 12 patients following a 3 + 3 algorithm to determine the maximum tolerated dose of aflibercept combined with docetaxel in patients with metastatic or unresectable solid tumors (trial registration: NCT00545246). Docetaxel (75 mg/m^2^ every 3 weeks or 60 mg/m^2^ after protocol amendment) was combined with escalating doses of aflibercept (2, 4 and 6 mg/kg every 4 weeks). Free and VEGF-bound aflibercept were measured to assess free aflibercept in excess of the VEGF-bound form. At the starting dose of the combination, 3 of 6 patients treated experienced febrile neutropenia. After reducing the docetaxel dose to 60 mg/m^2^ in step 2 and permitting therapeutic granulocyte colony-stimulating factor (G-CSF) use, 2 of 3 patients in both cohorts experienced febrile neutropenia. Five patients (42%) had a partial response and 4 patients had stable disease (33%). Free aflibercept in excess of the VEGF-bound form was not maintained at this dose level. The dose limiting toxicity (DLT) of aflibercept combined with docetaxel was febrile neutropenia, which occurred in 2 of 3 Japanese patients at the lowest aflibercept dose level (2 mg/kg) combined with docetaxel (60 mg/m^2^) and therapeutic G-CSF use. A recommended dose for further studies was not determined because of the DLT at the starting dose.

## Introduction

Several malignant tumors depend on angiogenesis, the growth of new blood vessels from the existing vasculature, to maintain a source of nutrition and oxygen from the body to support their growth and metastasis [1]. The process of angiogenesis, the outgrowth of new vessels from pre-existing vasculature, plays a central role in the development of this tumor blood supply. The control of angiogenesis is complex and involves multiple signaling pathways. Vascular endothelial growth factor (VEGF) is a powerful mitogen for endothelial cells that promotes the formation of new vessels required for normal and neoplastic tissue growth. In addition, VEGF potently increases vessel permeability. VEGF exerts its effect primarily through binding to receptor tyrosine kinases, VEGFR-1 (also called FLT1) and VEGFR-2 (also called KDR), expressed by endothelial cells [2, 3]. Clinical studies with the anti-VEGF monoclonal antibody, bevacizumab, have shown that targeting human VEGF-A is an effective treatment strategy in patients with metastatic colorectal cancer (mCRC) [4, 5], advanced non-small cell lung cancer (NSCLC) [6], metastatic renal cancer [7, 8] and glioblastoma multiforme [9].

Aflibercept is a soluble decoy receptor engineered to incorporate the second immunoglobulin (Ig)-like domain of VEGFR-1 joined to the third Ig-like domain of VEGFR-2 fused to the Fc portion of human IgG1 [10, 11]. This construction allows aflibercept to bind to all of the isoforms of VEGF-A with sub-picomolar affinity [12]. In addition, aflibercept is anticipated to be more active than other anti-VEGF agents because of its high binding affinity to VEGF and its ability to bind other related proangiogenic factors such as VEGF-B and the placental growth factors (PlGFs), PlGF1 and PlGF2 [13]. The large randomized, placebo-controlled, phase III VELOUR trial demonstrated that the addition of aflibercept to infusional 5-fluorouracil, folinic acid and irinotecan (FOLFIRI) significantly improved overall survival compared with placebo plus FOLFIRI in patients with mCRC previously treated with an oxaliplatin-based regimen [14]. Aflibercept was administered intravenously in VELOUR at a dose level of 4 mg/kg every 2 weeks.

The phase I dose-escalation study TCD6120 investigated aflibercept in combination with docetaxel in 54 predominantly Caucasian patients with advanced solid tumors [15]. Successive cohorts of patients received sequentially escalated doses of intravenous aflibercept (either 2, 4, 5, 6, 7 or 9 mg/kg) with docetaxel (75 mg/m^2^) on day 1 every 3 weeks until disease progression or unacceptable toxicity. The dose escalation was completed in this range of aflibercept doses, with 3 dose limiting toxicities (DLTs) at each dose level. In pharmacokinetics (PK) analysis, an excess of free-over-bound aflibercept was observed at doses of ≥ 5 mg/kg. Based on these data, the recommended dose of aflibercept for further investigation was 6 mg/kg in combination with docetaxel at 75 mg/m^2^ every 3 weeks. The primary objective of this study was to determine the recommended dose of aflibercept in combination with docetaxel for further studies in Japanese patients.

## Methods

### Patient eligibility

Patients aged ≥ 20 years were eligible if they had a histologically or cytologically confirmed solid malignancy that was metastatic or unresectable, for which docetaxel was appropriate. They must also have failed at least one prior line of standard treatment or been ineligible for standard care.

Patients were excluded if they met any of the following criteria: an Eastern Cooperative Oncology Group (ECOG) performance status > 1; unresolved (≥ grade 2) toxicity from prior anticancer therapy (excluding alopecia); inadequate bone marrow or organ function as evidenced by: hemoglobin < 9.0 g/dL; an absolute neutrophil count < 1.5 × 10^9^/L; platelets < 100 × 10^9^/L; creatinine > 1.0 × upper limit of normal (ULN; if between 1.0 to ≤ 1.5 × ULN, then the calculated creatinine clearance according to the Cockcroft-Gault formula was < 60 mL/min); urine protein: creatinine ratio > 1 and proteinuria > 500 mg/24 h; aspartate aminotransferase (AST) or alanine aminotransferase (ALT) > 2.5 × ULN (if alkaline phosphatase > 2.5 × ULN, then AST or ALT > 1.5 × ULN); total bilirubin > 1.0 × ULN; or serum albumin < 3.0 g/dL; a diagnosis of squamous-cell lung cancer; history of discontinuation of prior anti-VEGF therapy due to an adverse drug reaction; history of hypersensitivity to recombinant proteins, docetaxel or polysorbate 80; history of severe drug allergy; prior treatment with chemotherapy, hormonal therapy, radiotherapy, surgery, blood products or an investigational agent within the 28 days (42 days for nitrosourea agents, mitomycin C or immunotherapy) prior to study enrollment or cumulative radiation therapy to > 25% of the total bone marrow; history of brain metastases, spinal cord compression or carcinomatous meningitis, or new evidence of brain or leptomeningeal disease on screening computed tomography (CT) or magnetic resonance imaging (MRI) scan; peritoneal metastases clearly confirmed by CT or MRI; malignant ascites requiring drainage; active infection, hepatitis C virus, hepatitis B virus surface antigen positive or on antiviral therapy for human immunodeficiency virus; uncontrolled hypertension > 150/100 mmHg; severe cardiac, cerebral or gastrointestinal or thromboembolic events within 180 days prior to study entry; clinically significant bleeding diathesis or underlying coagulopathy; administration of warfarin; pregnant or breastfeeding; prior treatment with aflibercept; or prior discontinuation of docetaxel for safety reasons.

### Study design

This was a phase I, dual-center, open-label, dose-escalation study of aflibercept administered intravenously every 3 weeks in combination with docetaxel. The primary safety variable was DLT occurring during the first treatment cycle. Secondary variables were safety, PK, antitumor activity, and the immunogenicity of aflibercept. The protocol was approved by independent ethics committees at both participating centers and the study was conducted in accordance with the ethical principles laid out in the Declaration of Helsinki. All patients provided written informed consent prior to the initiation of any study-related procedures. The study was registered on ClinicalTrials.gov (NCT00545246; https://clinicaltrials.gov/ct2/show/NCT00545246).

### Drug dose and administration

The planned starting dose of aflibercept was 2 mg/kg every 3 weeks. Two further dose levels were planned: 4 mg/kg every 3 weeks and 6 mg/kg every 3 weeks. On day 1 of each 3-week cycle, aflibercept was to be administered intravenously over 1 h according to the assigned dose level followed by docetaxel, administered intravenously over ≥ 60 min, at 75 mg/m^2^ (or in the subsequent cohorts, at 60 mg/m^2^) in 250 mL 5% dextrose or 0.9% sodium chloride. Oral corticosteroids (i.e., dexamethasone 4 mg or 8 mg) were given pre- and post-administration of docetaxel. Prophylactic or therapeutic use of granulocyte colony-stimulating factor (G-CSF) was not permitted during the first cycle of study treatment unless the patient experienced a hematologic DLT.

Cohorts of 3–6 patients were to be treated at escalating dose levels of aflibercept, starting at 2 mg/m^2^, and the dose of docetaxel was held constant at 75 mg/m^2^. Dose escalation of aflibercept was based on the occurrence of DLTs during the first cycle following the 3 + 3 algorithm.

The maximum tolerated dose (MTD) of aflibercept combined with docetaxel was determined by following the 3 + 3 algorithm based on the occurrence of DLTs during the first cycle. To further explore the safety and preliminary efficacy profile of the recommended dose, the cohort of the recommended dose was planned to be expanded by up to 10 additional patients.

### Safety assessments and definition of DLT

Adverse events were graded according to the National Cancer Institute Common Terminology Criteria for Adverse Events version 3.0 (NCI CTCAE v.3.0). DLTs included any of the following adverse events during the first treatment cycle: grade 3 or 4 neutropenia complicated by fever (≥ 38.5 °C) or infection; grade 4 neutropenia lasting > 7 days; grade 4 thrombocytopenia, or grade 3 thrombocytopenia complicated by hemorrhage; any grade 3 non-hematologic adverse event except fatigue, anorexia, nausea, vomiting, hyponatremia (unless such inclusion was subsequently deemed necessary; for example, if these adverse events were excessive in frequency or duration, or required excessive use of supportive therapy); any grade 4 non-hematologic adverse event; uncontrolled hypertension as defined by systolic blood pressure (BP) > 150 mmHg or diastolic BP > 100 mmHg (or > 180/90 mmHg if the patient had a history of isolated systolic hypertension) despite 4 weeks of medical management; urinary protein excretion of > 3.5 g per 24 h that does not recover to < 2.0 g per 24 h within 2 weeks; symptomatic arterial thromboembolic events including cerebrovascular accident, myocardial infarction, transient ischemic attack, and new onset or worsening of pre-existing angina.

### Efficacy assessment

Following RECIST guidelines, tumor responses were assessed by CT or MRI on day 21 of every cycle or to confirm a partial or complete response (4–6 weeks after the initial documentation of response), whenever disease progression was suspected, and at the end of study treatment [16]. Antitumor efficacy was also evaluated by positron emission tomography (PET) at baseline, on day 21 of cycle 1, and then on day 21 of every even-numbered cycle.

### PK and immunogenicity

Blood samples (4.5 mL) were collected for the analysis of plasma concentrations of free and VEGF-bound aflibercept before the start of aflibercept infusion on day 1 of cycle 1 (predose); at the end of infusion; and 1, 3, 7, 23, 29, 47, 167 (day 7) and 335 h (day 14) after the end of infusion. After cycle 2, blood samples were collected before each aflibercept infusion, and 30 and 90 days after the last aflibercept administration. Free and VEGF-bound aflibercept were measured in plasma using a validated direct enzyme-linked immunosorbent assay (ELISA). The concentrations of VEGF-bound aflibercept were converted to free aflibercept equivalents (adjusted values) based on the molecular weights of VEGF and aflibercept for PK analysis. The lower limit of quantification (LLOQ) was 0.0156 µg/mL and 0.0315 µg/mL (adjusted) for free and VEGF-bound aflibercept, respectively. PK parameters were calculated by noncompartmental analysis on a validated PK data management system using WinNonlin Professional, Version 5.2.1 (Pharsight).

Blood samples (1 mL) were taken to determine the docetaxel plasma concentrations before the start of aflibercept infusion on day 1 of cycle 1, 30 min after the start of the docetaxel infusion, immediately before the end of the docetaxel infusion, and 10 min, and 2, 5, 7 and 24 h after the end of the docetaxel infusion. Plasma samples were analyzed using a validated liquid chromatography and tandem mass spectrometry method with an LLOQ of docetaxel of 1 ng/mL. The PK parameters for each patient were estimated using a Bayesian estimation method and the adult population PK model as prior information [17].

To screen for the presence of anti-aflibercept antibodies in serum, blood samples (4.0 mL) were collected predose on day 1 of every odd-numbered cycle, upon study withdrawal, and 90 days after study treatment discontinuation. Antibody levels were measured using a validated ELISA method with an LLOQ of 52.7 IU/mL.

## Results

### Patients

Twelve Japanese patients were included. Their baseline characteristics are summarized in Table 1. The patients were predominantly female; the median age was 63.5 years, and all had an ECOG performance status of 0 or 1. The most common primary tumor sites were lung, ovary and breast. The patients had received a median of 2.5 lines of prior chemotherapy (range 1–6). All 12 enrolled patients were evaluable for safety, PK and efficacy.

**Table 1:** Baseline patient and disease characteristics

**Characteristic**	**Dose level**^a^			**All patients**
	**A2D75**	**A2D60**	**A2D60G**	
	**(N = 6)**	**(N = 3)**	**(N = 3)**	**(N = 12)**
Sex, n (%)				
Female	4 (67)	3 (100)	2 (67)	9 (75)
Male	2 (33)	0	1 (33)	3 (25)
Age, years				
Median	60.0	65.0	56.0	63.5
Range	55–74	63–68	51–72	51–74
Weight, kg				
Median	51.45	53.00	49.10	51.05
ECOG PS, n (%)				
0	4 (67)	0	2 (67)	6 (50)
1	2 (33)	3 (100)	1 (33)	6 (50)
Primary tumor site, n (%)				
Lung	1 (17)	2 (67)	1 (33)	4 (33)
Ovary	3 (50)	0	0	3 (25)
Breast	0	0	2 (67)	2 (17)
Other^b^	2 (33)	1 (33)	0	3 (25)
Prior anticancer therapy,^c^ n (%)				
Chemotherapy	6 (100)	3 (100)	3 (100)	12 (100)
Surgery	5 (83)	2 (67)	2 (67)	9 (75)
Radiotherapy	2 (33)	0	2 (67)	4 (33)
Number of lines of prior chemotherapy				
Median	3.5	2.0	1.0	2.5
Range	1–6	1–4	1–6	1–6

^a^A2D75 = aflibercept 2 mg/kg + docetaxel 75 mg/m^2^; A2D60 = aflibercept 2 mg/kg + docetaxel 60 mg/m^2^; A2D60G = aflibercept 2 mg/kg + docetaxel 60 mg/m^2^ (+ granulocyte colony-stimulating factor, if necessary)

^b^A2D75, lung/prostate (1 patient), fallopian tube (1 patient); A2D60, ovary/rectum

^c^A patient may have received more than one type of prior anticancer therapy

*ECOG PS* Eastern Cooperative Oncology Group performance status

### Safety and tolerability

Three out of 6 patients treated at the starting dose level (aflibercept 2 mg/kg plus docetaxel 75 mg/m^2^; A2D75) experienced a DLT of grade 3 febrile neutropenia without prophylactic or therapeutic use of G-CSF. Because it was considered that this DLT was mainly caused by docetaxel, the study protocol was amended to reduce the docetaxel dose to 60 mg/m^2^ in combination with aflibercept at the starting dose (A2D60). In this subsequent cohort, 2 of the 3 patients experienced a DLT (in both cases, grade 3 febrile neutropenia). A second protocol amendment was therefore implemented by allowing the therapeutic use of G-CSF to manage febrile neutropenia if grade 4 neutropenia, or grade 3 neutropenia complicated by a temperature ≥ 38 °C, were observed. However, 2 of 3 patients treated at the lowest aflibercept dose level (2 mg/kg; A2D60G) experienced a DLT (grade 3 febrile neutropenia) despite use of G-CSF.

Although febrile neutropenia occurred as a DLT in 7 of 12 enrolled patients, none of these patients withdrew from study treatment. Patients in the A2D75 (n = 6), A2D60 (n = 3) and A2D60G (n = 3) dose level cohorts received a median of 6, 10 and 30 treatment cycles, respectively, with a total of 36, 29 and 69 cycles, respectively. The median durations of each cycle were 3.93, 4.00 and 4.00 weeks, respectively. Ten of 12 patients discontinued study treatment due to disease progression. One patient in the A2D60 group discontinued study treatment due to a treatment emergent adverse event (AE; grade 2 sensory disturbance unrelated to aflibercept) and 1 patient in the A2D75 group discontinued because sustained treatment was deemed detrimental to the patient’s well-being.

The most commonly reported AEs at any grade were neutropenia, alopecia, fatigue, febrile neutropenia, decreased appetite, and stomatitis, each of which occurred in ≥ 10 patients overall (Table 2). The most common grade 3/4 AEs were neutropenia and febrile neutropenia, which were reported for 12 and 10 patients, respectively. No other grade 3/4 AE occurred in more than 1 patient.

**Table 2:** Incidence of the most common treatment emergent adverse events^a^

**Preferred term**^**b**^** n (%)**	**Dose level**^c^						**All patients**	
	**A2D75** **(N = 6)**		**A2D60** **(N = 3)**		**A2D60G** **(N = 3)**		**(N = 12)**	
	All grades	Grade 3/4	All grades	Grade 3/4	All grades	Grade 3/4	All grades	Grade 3/4
Neutropenia	6 (100)	6 (100)	3 (100)	3 (100)	3 (100)	3 (100)	12 (100)	12 (100)
Alopecia	6 (100)	0	3 (100)	0	3 (100)	0	12 (100)	0
Fatigue	6 (100)	0	3 (100)	0	2 (67)	0	11 (92)	0
Febrile neutropenia	6 (100)	6 (100)	2 (67)	2 (67)	2 (67)	2 (67)	10 (83)	10 (83)
Decreased appetite	6 (100)	1 (17)	2 (67)	0	2 (67)	0	10 (83)	1 (8)
Stomatitis	5 (83)	0	2 (67)	0	3 (100)	0	10 (83)	0
Dysphonia	3 (50)	0	3 (100)	0	3 (100)	0	9 (75)	0
Flushing	5 (83)	0	2 (67)	0	2 (67)	0	9 (75)	0
Pyrexia	3 (50)	0	3 (100)	0	3 (100)	0	9 (75)	0
Diarrhea	3 (50)	0	2 (67)	0	3 (100)	0	8 (67)	0
Dysgeusia	3 (50)	0	2 (67)	0	3 (100)	0	8 (67)	0
Hypertension	4 (67)	0	2 (67)	1 (33)	1 (33)	0	7 (58)	1 (8)
Arthralgia	4 (67)	0	1 (33)	0	2 (67)	0	7 (58)	0
Epistaxis	3 (50)	0	2 (67)	0	2 (67)	0	7 (58)	0
Insomnia	3 (50)	0	3 (100)	0	1 (33)	0	7 (58)	0
Nausea	3 (50)	0	2 (67)	0	2 (67)	0	7 (58)	0
Vomiting	4 (67)	0	1 (33)	0	2 (67)	0	7 (58)	0
Nail disorder	3 (50)	0	2 (67)	0	2 (67)	0	7 (58)	0

^a^Reported in > 6 patients overall at any grade

^b^Adverse events are reported according to the Medical Dictionary for Regulatory Activities version 15.0

^c^A2D75 = aflibercept 2 mg/kg + docetaxel 75 mg/m^2^; A2D60 = aflibercept 2 mg/kg + docetaxel 60 mg/m^2^; A2D60G = aflibercept 2 mg/kg + docetaxel 60 mg/m^2^ (+ granulocyte colony-stimulating factor, if necessary)

### PK and immunogenicity

**Fig. 1:**
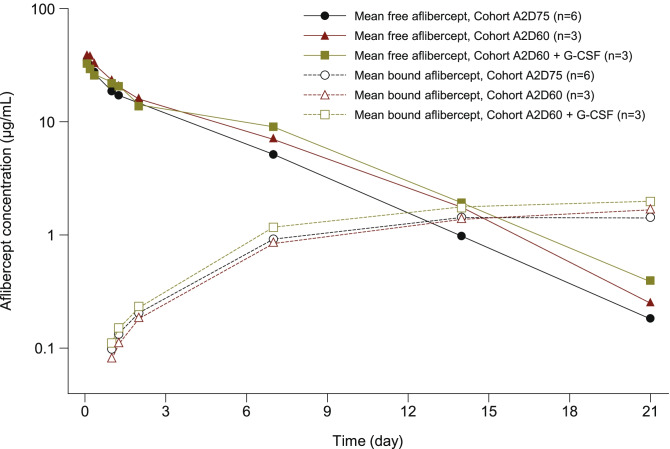
Mean free and VEGF-bound aflibercept concentration–time profiles across cycle 1 (semi-log scale)

**Fig. 2:**
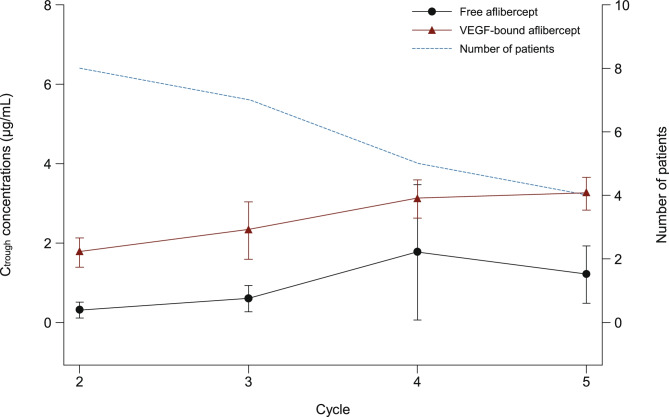
Mean (± standard deviation) free and VEGF-bound aflibercept C_trough_ values across cycles

The PK parameters for free and VEGF-bound aflibercept are summarized in Table 3. The mean T_max_ for free aflibercept was 0.08 days. Free aflibercept had a terminal half-life of approximately 3 days and was eliminated with clearance of 0.8 L/day. The volume of distribution at steady state was 3.4 L. The mean free and VEGF-bound plasma concentrations across cycle 1 for each dose level cohort are illustrated in Fig. 1. The mean VEGF-bound aflibercept concentrations for each cohort increased across the first cycle to reach a maximum on day 21 post-dose. In contrast, the mean concentrations of free aflibercept fell across cycle 1, dropping below the levels of VEGF-bound aflibercept between days 12 and 15 (Fig. 1). Up to cycle 4, the free and VEGF-bound C_trough_ levels of aflibercept increased across cycles (Fig. 2). However, there were insufficient data to determine whether steady state was reached by cycle 5. The individual and mean values of the free to VEGF-bound C_trough_ ratio during repeated administrations of aflibercept at 2 mg/kg every 3 weeks were generally below 1.

**Table 3:** Plasma pharmacokinetic parameters of free and VEGF-bound aflibercept in cycle 1 following administration at 2 mg/kg

**Mean ± SD (CV%) parameters**	**Free aflibercept**	**VEGF-bound aflibercept**
Number of patients	12	
C_max_, μg/mL	36.8 ± 9.72 (26)	1.72 ± 0.343 (20)
T_max,_^a^ day	0.08 (0.04–0.17)	20.99 (14.00–28.01)
AUC_last_, μg·day/mL	131 ± 24.8 (19)	24.7 ± 6.39 (26)
AUC_0–21 day_, μg·day/mL	131 ± 23.7 (18)	22.7 ± 3.39^b^ (15)
AUC, μg·day/mL	133 ± 25.1 (19)	-
t_1/2z_, day	3.23 ± 0.495 (15)	-
CL, L/day	0.801 ± 0.144 (18)	-
V_ss_, L	3.44 ± 0.558 (16)	-

^a^Median (range)

^b^N = 11 (one patient was not evaluable)

*SD* standard deviation, *CV%* coefficient of variation percentage, *C*_*max*_ maximum drug concentration observed, *T*_*max*_ first time to reach C_max_, *AUC*_*last*_ area under the concentration versus time curve calculated from time zero to the real time T_last_, *AUC*_*0–21 day*_ area under the concentration versus time curve from time zero to day 21, *AUC* area under the concentration versus time curve extrapolated to infinity, *t*_*1/2z*_ terminal elimination half-life, *CL* apparent total body clearance of drug from the plasma, *V*_*ss*_ volume of distribution at steady state

The mean (± standard deviation) docetaxel CL was 21.7 ± 4.01 (range: 16.1–30.6) L/h/m^2^ corresponding to a mean AUC of 3.60 ± 0.524 (range: 2.99–4.30) μg·day/mL for patients receiving a 75 mg/m^2^ dose and to a mean AUC of 2.85 ± 0.621 (range: 2.00–3.79) μg·day/mL for patients receiving a 60 mg/m^2^ dose.

All 12 patients treated with aflibercept were evaluable for immunogenicity and found to be negative for anti-aflibercept antibodies.

### Antitumor activity

Tumor response was evaluated according to RECIST in all patients. Five of 12 patients (42%) had a partial response, including 1 (lung/prostate for 9 cycles) of 6 in the A2D75 cohort, 2 (both lungs for 16 and 10 cycles) of 3 in the A2D60 cohort, and 2 (both breasts for 30 and 35 cycles) of 3 in the A2D60G cohort. The primary tumor sites of the 5 patients were lung (n = 3) and breast (n = 2). Four patients had stable disease, including 2 in the A2D75 cohort, 1 in the A2D60 cohort, and 1 in the A2D60G cohort. Two patients, both in the A2D75 cohort, had a best overall response of progressive disease. The response status was not assessed in 1 patient who discontinued treatment in cycle 1 in the A2D75 cohort.

According to the PET assessment, 8 of 12 evaluable patients had an effective response to treatment, including 3 in the A2D75 cohort, 2 in the A2D60 cohort, and 3 in the A2D60G cohort. Four patients had no change, including 3 in the A2D75 cohort and 1 in the A2D60 cohort.

## Discussion

The objective of this study was to determine a recommended dose of aflibercept that could be safely administered in combination with docetaxel in Japanese patients with advanced solid malignancies. Febrile neutropenia was observed as DLT in 3 of the 6 patients treated at the lowest dose level (2 mg/kg aflibercept plus docetaxel 75 mg/m^2^) evaluated in the initial protocol. Because severe neutropenia was a common side effect associated with docetaxel administration in Japanese patients [18–22], we decided to reduce the planned docetaxel dose level from 75 mg/m^2^ to 60 mg/m^2^. Despite this reduction, febrile neutropenia was observed as a DLT in 2 of 3 patients in this cohort. A subsequent protocol modification allowed the therapeutic use of G-CSF in cycle 1 under certain specific conditions. However, this did not appear to resolve the occurrence of febrile neutropenia, which was reported as a DLT in 2 of 3 patients at the lowest aflibercept dose level. In view of the incidence of this DLT, we could not determine a recommended dose of aflibercept combined with docetaxel for use in further studies in Japanese patients. The combination of aflibercept 2 mg/kg and docetaxel 60 mg/m^2^ with therapeutic G-CSF was not tolerable in the dose-finding study in Japanese patients.

The combination of aflibercept (6 mg/kg) and docetaxel (75 mg/kg) was previously investigated in the large multinational phase III VITAL study, in which 913 patients with platinum-pretreated NSCLC were randomized to docetaxel with either aflibercept or placebo [23]. Patients were enrolled from 162 centers in 29 countries, although there was no recruitment in Japan. Although the rate of grade ≥ 3 neutropenia was higher in the aflibercept/docetaxel arm compared with the placebo/docetaxel arm (28.0% vs 21.1%, respectively), the incidence of febrile neutropenia was relatively low (6.6% vs 4.2%, respectively). A recent phase I study in Japanese patients with mCRC previously showed the combination of aflibercept at 4 mg/kg and FOLFIRI to be well tolerated [24]. In the 16 patients included at aflibercept dose levels of 2 mg/kg (n = 3) or 4 mg/kg (n = 13), febrile neutropenia was not reported as a DLT for any patient in the first 2 cycles. The explanation for the relatively high incidence of febrile neutropenia seen in this study (7 of 12 patients; 58%) is therefore not immediately obvious. In particular, the PK parameters for aflibercept were comparable with those previously reported for non-Japanese [15, 25] and Japanese [24] patient cohorts receiving 2 mg/kg in other phase I studies. In addition, the mean CL and AUC values for docetaxel 75 mg/m^2^ in Japanese patients were similar to those previously reported for Caucasian patients [26].

Studies in murine model systems have suggested that in order to achieve maximum antitumor activity, the level of free aflibercept in plasma should exceed the level of VEGF-bound aflibercept over the cycles [27]. Due to the incidence of DLT, it was not possible to escalate the dose level above 2 mg/kg in the present study. At this dose, VEGF-bound aflibercept did not reach a plateau level and an excess of free over VEGF-bound aflibercept was not maintained across cycles. The aflibercept dose of 2 mg/kg every 3 weeks combined with docetaxel did not achieve the target level of pharmacological exposure in Japanese patients. However, of 12 patients treated in this study, 5 had a partial response to the treatment and 4 patients had stable disease. In addition, although the incidence of febrile neutropenia was relatively high, none of the patients withdrew from study treatment. These results suggest that aflibercept combined with docetaxel may have some therapeutic potential in Japanese patients, and that prophylactic G-CSF administration should be mandatory when using this combination.

In many tumor types, chemotherapy-induced severe neutropenia is associated with improved overall survival [28, 29]. In a large pooled analysis of 1529 patients with NSCLC treated with chemotherapy in 6 randomized studies, chemotherapy-induced neutropenia was significantly associated with a longer overall survival, especially in patients who developed severe neutropenia [30]. Similar findings were reported in pancreatic cancer [31], gastric cancer [32], and metastatic castration-resistant prostate cancer [33]. There is increasing evidence that tumor-associated myeloid cells play a crucial role in tumor development, metastatic progression, and the immunosuppressive microenvironment of many cancers [34, 35]. Tumor-associated myeloid cells also induce resistance to anti-angiogenic drugs [36]. Taxanes, by inducing neutropenia, may contribute to delaying tumor progression and prolonged survival, and thus restore sensitivity to anti-angiogenics in some types of cancers [33].

In conclusion, the DLT of aflibercept in combination with docetaxel was febrile neutropenia in Japanese patients. A recommended dose for further investigation was not determined due to the incidence of DLTs at the starting dose. However, the encouraging antitumor activity observed in these patients deserves further investigation.

## Data Availability

Qualified researchers can request access to patient-level data and related study documents including the clinical study report, study protocol with any amendments, blank case report forms, statistical analysis plan, and dataset specifications. Patient-level data will be anonymized, and study documents will be redacted to protect the privacy of trial participants. Further details on Sanofi’s data-sharing criteria, eligible studies, and process for requesting access are at: https://www.vivli.org/.
